# Spermatological Characterization of the Cestode *Meggittina gerbilli* (Cyclophyllidea: Catenotaeniidae), a Parasite of Gerbils, *Gerbillus gerbillus* and *Gerbillus campestris* (Rodentia: Muridae) in Tunisia

**DOI:** 10.3390/ani14010012

**Published:** 2023-12-19

**Authors:** Faouzi Aouina, Hichem Kacem, Natalia Martín-Carrillo, Pilar Foronda, Jordi Miquel

**Affiliations:** 1 Laboratoire Écologie et Environement, Faculté des Sciences de Gabès, Université de Gabès Zrig, Gabès 6072, Tunisia; faouzi_aouina@yahoo.fr (F.A.); hichem.kacem@fss.usf.tn (H.K.); 2 Département des Sciences de la Vie, Faculté des Sciences de Sfax, Université de Sfax, Sfax 3000, Tunisia; 3 Instituto Universitario de Enfermedades Tropicales y Salud Pública de Canarias, Universidad de La Laguna, Av. Astrofísico F. Sánchez, sn, 38203 La Laguna, Spain; nmartinc@ull.edu.es (N.M.-C.); pforonda@ull.edu.es (P.F.); 4 Departamento Obstetricia y Ginecología, Pediatría, Medicina Preventiva y Salud Pública, Toxicología, Medicina Legal y Forense y Parasitología, Facultad de Farmacia, Universidad de La Laguna, Av. Astrofísico F. Sánchez, sn, 38203 La Laguna, Spain; 5 Secció de Parasitologia, Departament de Biologia, Sanitat i Medi Ambient, Facultat de Farmàcia i Ciències de l’Alimentació, Universitat de Barcelona, Av. Joan XXIII, sn, 08028 Barcelona, Spain; 6 Institut de Recerca de la Biodiversitat (IRBio), Universitat de Barcelona, Av. Diagonal, 645, 08028 Barcelona, Spain

**Keywords:** sperm characters, ultrastructure, *Meggittina gerbilli*, Cestoda, Cyclophyllidea, Catenotaeniidae, *Gerbillus gerbillus*, *Gerbillus campestris*, Muridae, Tunisia

## Abstract

**Simple Summary:**

Ultrastructural characters of spermiogenesis and the spermatozoon of the cestode *Meggittina gerbilli*, a parasite of gerbils in Tunisia, were studied using transmission electron microscopy. The type III model of spermiogenesis was observed. This model is mainly characterized by a proximodistal fusion of a single flagellum with a cytoplasmic extension. As for the sperm cell, spermatozoon type VI was observed, presenting a single axoneme, a periaxonemal sheath, crest-like bodies, twisted cortical microtubules, and a spiraled nucleus. The results show similarities between *Meggittina gerbilli* and other studied species within the Catenotaeniidae family.

**Abstract:**

Ultrastructural characters of spermiogenesis and the mature spermatozoon of the cestode *Meggittina gerbilli* (Cyclophyllidea: Catenotaeniidae), a parasite of the Lesser Egyptian gerbil (*Gerbillus gerbillus*) and the North African gerbil (*Gerbillus campestris*) (Rodentia: Muridae) in the Djebel Dahar (South of Tunisia), were studied using transmission electron microscopy. The spermiogenesis of *M. gerbilli* is of Bâ and Marchand’s type III, which is mainly characterized by a proximodistal fusion of a single flagellum with a cytoplasmic extension. In this catenotaeniid, the proximal fusion is preceded by a 90° rotation of the flagellum. The spermatozoon is a Levron et al. type VI, which presents a single axoneme with the 9 + ‘1’ trepaxonematan pattern, a periaxonemal sheath, two crest-like bodies, twisted cortical microtubules, and a spiraled nucleus. The obtained results show similarities with the remaining studied catenotaeniids, namely *Catenotaenia pusilla* and *Skrjabinotaenia lobata*. The results are compared and discussed according to several characteristics found in the catenotaeniids and other studied cyclophyllideans.

## 1. Introduction

The genus *Meggittina* Lynsdale, 1953 includes catenotaeniid cestodes parasites of Muridae and Nesomyidae rodents in Africa [1,2], although some records concern murids from Israel [3,4]. Currently, this genus is included in the subfamily Skrjabinotaeniinae Genov and Tenora, 1979 and has four valid species, namely *Meggittina baeri* Lynsdale, 1953, *Meggittina gerbilli* (Wertheim, 1954), *Meggittina aegyptica* (Wolfgang, 1956), and *Meggittina cricetomydis* (Hockley, 1961) [5]. The genus *Meggittina* and the species *M. baeri* were erected by Lynsdale [6] using specimens recovered from unidentified rat species (“house rat” and “native granary rat”) in Southern Rhodesia (Zimbabwe). After the original description, *M. baeri* was reported in *Cricetomys gambianus* Waterhouse, 1840 from Nigeria [7], in *Eliurus talala* Major, 1896 from Madagascar [1], and *Rhabdomys pumilio* (Sparrman, 1784) from South Africa [2]. Several taxonomy problems concerning the genus *Meggittina* have evolved over the years. For example, in addition to the four currently valid species, another species, *Meggittina numida* Jrijer and Neifar, 2014 was described in Tunisia as a parasite of *Meriones shawi* (Duvernoy, 1842) [8]. However, in a recent phylogenetic analysis of the family Catenotaeniidae Spassky, 1950, Haukisalmi et al. [2] considered this species a junior synonym of *Skrjabinotaenia oranensis* (Joyeux and Foley, 1930). *Meggittina aegyptica* was originally described as *Catenotaenia aegyptica* Wolfgang, 1956 parasitizing *Meriones* sp., *Acomys cahirinus* (Geoffroy, 1803) and *Gerbillus gerbillus* (Olivier, 1801) in Egypt [9], and recently, it was cited in Tunisia in *M. shawi* [10]. *Meggittina cricetomydis* was described as *Skrjabinotaenia cricetomydis* Hockley, 1961 in *C. gambianus* in Nigeria [11], and later reported in the same host and country by Jones [12], who confirmed its inclusion in the genus *Meggittina*. Finally, *M. gerbilli* was described as *Rajotaenia gerbilli* Wertheim, 1954 parasitizing *Gerbillus pyramidum* Geoffroy, 1825 in Israel [3]. Posteriorly, the species was found in *Gerbillus dasyurus* Wagner, 1842, *G. gerbillus*, *G. pyramidum*, and *Meriones crassus* Sundevall, 1842 in Israel and Egypt [4], and in *M. shawi* in Tunisia [13]. In a systematic study of the suborder Catenotaeniata Spassky, 1963, Tenora et al. [14] synonymized the genus *Rajotaenia* Wertheim, 1954 with *Meggittina*.

Ultrastructural studies on spermiogenesis and the spermatozoon have been demonstrated as a useful source of characters for phylogenetic inference in diverse parasitic groups of Platyhelminthes [15,16,17,18,19,20,21,22,23,24,25]. In the case of cestodes, Świderski [16] described three models of spermiogenesis, namely the pseudophyllidean type, the caryophyllidean type, and the cyclophyllidean type, which were based on the number of flagella/axonemes that originated from the differentiation zone and the presence/absence of the flagella proximodistal fusion. Bâ and Marchand [20] added a fourth model to differentiate spermiogenesis originating a single free flagellum, according to whether the flagellum develops orthogonal or parallel to the cytoplasmic process (spermiogenesis type II or III, respectively). Thus, Bâ and Marchand’s type I corresponds to the pseudophyllidean model of Świderski, type II to the caryophyllidean model, and type IV to the cyclophyllidean model. The new Bâ and Marchand’s type III spermiogenesis is also found in cyclophyllidean cestodes. According to the latter authors, the most evident difference between types III and IV, exhibited by cyclophyllideans, is the presence/absence of proximodistal fusion. Thus, in type III spermiogenesis, a flagellum grows externally and parallel to the cytoplasmic process, which is followed by proximodistal fusion. In type IV, an axoneme grows directly into the cytoplasmic process; thus, there is no proximodistal fusion. Justine [21] established several synapomorphies on the basis of ultrastructural characters of spermiogenesis and the spermatozoon for diverse orders of the Eucestoda, e.g., the absence of mitochondrion for the Eucestoda or the spiraled pattern of cortical microtubules for the tetrabothriideans and cyclophyllideans, among others. Finally, considering the available spermatological studies at the time, Levron et al. [23] established seven spermatozoon models in the Eucestoda (I to VII). Types I and II spermatozoa are mainly characterized by having two axonemes, while they are differentiated by the presence/absence of crest-like bodies. The remaining models of sperm cells (types III to VII) have a single axoneme, and they can be distinguished by considering the presence/absence of other characters, such as crest-like bodies, periaxonemal sheath, and intracytoplasmic walls, and the parallel or spiraled pattern of cortical microtubules and nucleus.

In the Cestoda, there are numerous ultrastructural studies describing spermiogenesis and/or the spermatozoon, particularly in the order Cyclophyllidea [23,26,27,28,29,30,31,32]. However, in the Catenotaeniidae family, ultrastructural aspects of spermiogenesis and/or the spermatozoon have only been studied in two species, namely *Catenotaenia pusilla* (Goeze, 1782) (Catenotaeniinae Spassky, 1950) and *Skrjabinotaenia lobata* (Baer, 1925) (Skrjabinotaeniinae) [33,34]. The subfamily Skrjabinotaeniinae has only two genera, namely *Skrjabinotaenia* Akhumyan, 1946 and *Meggittina*, and to date, no ultrastructural information on the genus *Meggittina* is available. In the present study, we described the ultrastructural characteristics of spermiogenesis and the spermatozoon in the cestode *Meggittina gerbilli*, a parasite of the gerbils *Gerbillus gerbillus* and *Gerbillus campestris* (Levaillant, 1857) in Tunisia.

## 2. Materials and Methods

### 2.1. Specimens

**Figure 1:**
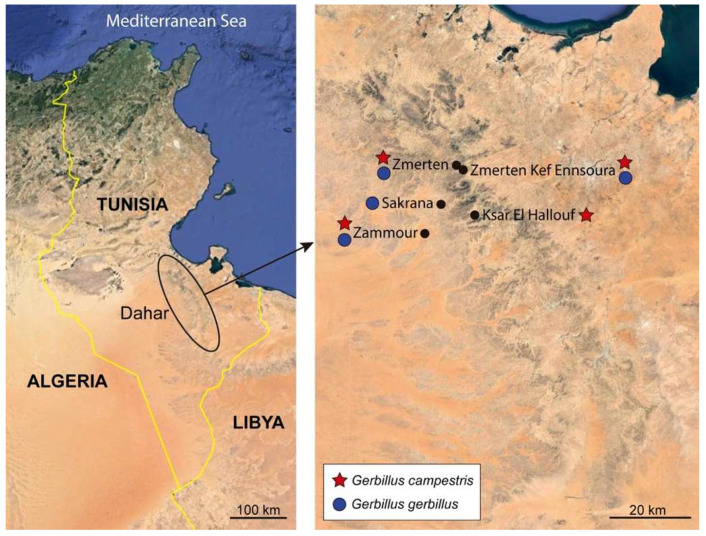
Sampling locations for North African gerbils and lesser Egyptian gerbils containing the parasite *Meggittina gerbilli* in the Djebel Dahar (Tunisia). Images captured from Google Earth Pro and modified using Adobe Illustrator 2024 software (Adobe, San José, CA, USA).

Specimens of the cestode *Meggittina gerbilli* (Cyclophyllidea, Catenotaeniidae) were recovered from the intestinal tract of 11 naturally infected gerbils captured in the Djebel Dahar (South of Tunisia) in March, April, and June 2023. Of the 11 infected gerbils, seven were North African gerbils *G. campestris*, which had been trapped in Ksar El Hallouf, Zammour, Zmerten, and Zmerten Kef Ennsoura, while the remaining four were lesser Egyptian gerbils *G. gerbillus*, which had been trapped in Sakrana, Zammour, Zmerten, and Zmerten Kef Ennsoura (Figure 1). The studied gerbils were captured using Manufrance-type (Saint-Étienne, France) or Firobind-type (Besançon, France) wire-mesh traps and sacrificed by cervical dislocation before being examined for intestinal helminths using a stereomicroscope.

### 2.2. Species Identification

**Figure 2:**
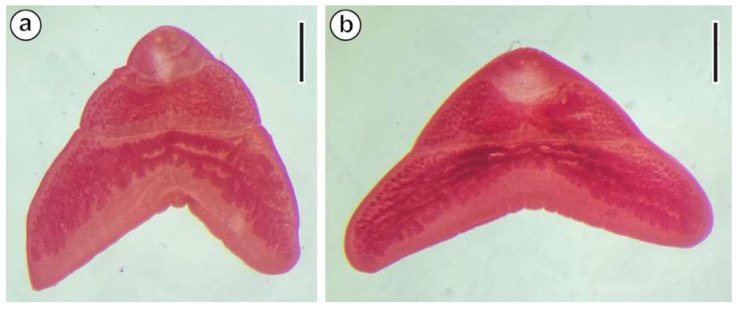
*Meggittina gerbilli* stained with Semichon’s acetic carmine. (**a**) Specimen no. 23031803 ex. *G. gerbillus* from Zammour. (**b**) Specimen no. 23031901 ex. *G. campestris* from Zammour. Scale bars = 1 mm.

Several cestodes were fixed in the field in ethanol at 70% and later, in the laboratory, they were stained with Semichon’s acetic carmine, dehydrated in an ethanol series and with 2-propanol, cleared in clove oil, and, finally, mounted on slides with Canada balsam (Figure 2). Specimens were identified as *M. gerbilli*, in agreement with specialized literature [3,6,8,9], using a Leica DMLB light microscope (Leica Microsystems, Wetzlar, Germany) at magnifications of 100× and 400×.

Two slides with four specimens of *M. gerbilli* ex. *G. gerbillus* and *G. campestris* from Zmerten (Tunisia) were deposited in the “Muséum National d’Histoire Naturelle” (Paris, France) under the accession numbers MNHN HEL2056 (no. 23041502 ex. *G. campestris*, 33°25′4.48″ N, 10°7′54.23″ E) and MNHN HEL2057 (no. 23041503 ex. *G. gerbillus*, 33°25′6.93″ N, 10°7′55.73″ E).

### 2.3. Transmission Electron Microscopy Study

**Figure 3:**
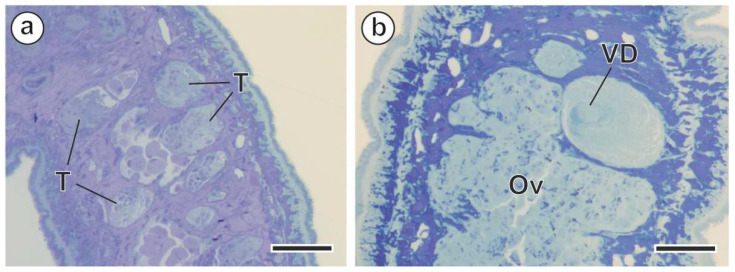
Semithin sections of *Meggittina gerbilli* were stained with 1% methylene blue–1% borax. (**a**) Semithin section of testes. (**b**) Semithin section of the vas deferens. (Ov) ovary; (T) testes; (VD vas deferens. Scale bars = 50 µm.

Some living adult cestodes were rinsed with a 0.9% NaCl solution immediately upon removal from the intestinal tract of both gerbil species. Then, they were fixed in 2.5% glutaraldehyde at 4 °C in a 0.1 M sodium cacodylate buffer (pH 7.4) for a minimum of 2 h. After rinsing in a 0.1 M sodium cacodylate buffer (pH 7.4), the specimens were postfixed in 1% osmium tetroxide at 4 °C with 0.9% potassium ferricyanide in the same buffer for 1 h. After rinsing in Milli-Q water (Millipore Gradient A10, Millipore Co., Merck KGaA, Darmstadt, Germany), the dehydration process was started by using an ethanol series and propylene oxide. The specimens were finally embedded in Spurr’s epoxy resin and polymerized at 60 °C for 72 h. Semithin sections (1 µm thick) were obtained using a Leica Reichert–Jung Ultracut E ultramicrotome (Leica Microsystems, Wetzlar, Germany), placed on slides, and stained with a mixture of 1% methylene blue–1% borax. Semithin sections were used to locate the study area (testes and vas deferens) (Figure 3). Ultrathin sections (60 nm thick) were obtained using a Leica Reichert–Jung Ultracut E ultramicrotome (Leica Microsystems), placed on gold grids, and double-stained with uranyl acetate and lead citrate, as in Reynolds [35]. Stained ultrathin sections were observed under a JEOL 1010 transmission electron microscope (JEOL Ltd., Tokyo, Japan) operated at an accelerating voltage of 80 kV in the “Centres Científics i Tecnològics de la Universitat de Barcelona (CCiTUB)”.

## 3. Results

### 3.1. Spermiogenesis 

**Figure 4:**
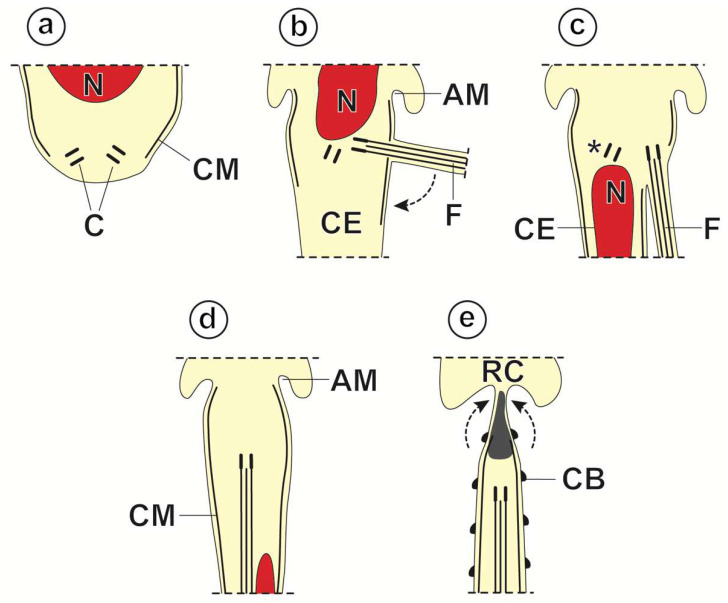
(**a**–**e**). Schematic drawing showing the main stages of spermiogenesis in *Meggittina gerbilli*. (AM) arching membranes; (C) centrioles; (CB) crest-like bodies; (CE) cytoplasmic extension; (CM) cortical microtubules; (F) flagellum; (N) nucleus; (RC) residual cytoplasm; (*) abortive centriole.

**Figure 5:**
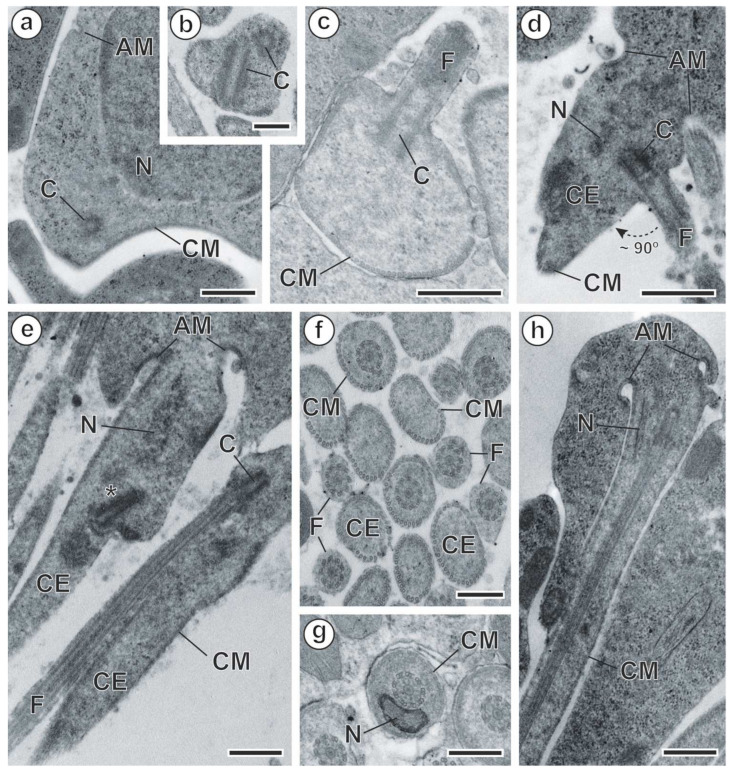
Spermiogenesis of *Meggittina gerbilli*. (**a**) Differentiation zone showing one of the two centrioles. (**b**) Detail of the two orthogonally placed centrioles. (**c**,**d**) Cross- and longitudinal sections showing the flagellum growing orthogonally to the cytoplasmic extension. (**e**) Spermatids after flagellar rotation. (**f**) Cross-sections showing spermatids before and after the proximodistal fusion between the free flagellum and the cytoplasmic extension. (**g**) Cross-section showing the nucleus. (**h**) A longitudinal section of a spermatid showing the nucleus migration. (*) abortive centriole; (AM) arching membrane; (C) centriole; (CE) cytoplasmic extension; (CM) cortical microtubules; (F) flagellum; (N) nucleus. Scale bars: (**a**,**c**–**e**,**h**) = 500 nm, (**b**,**f**,**g**) = 300 nm.

**Figure 6:**
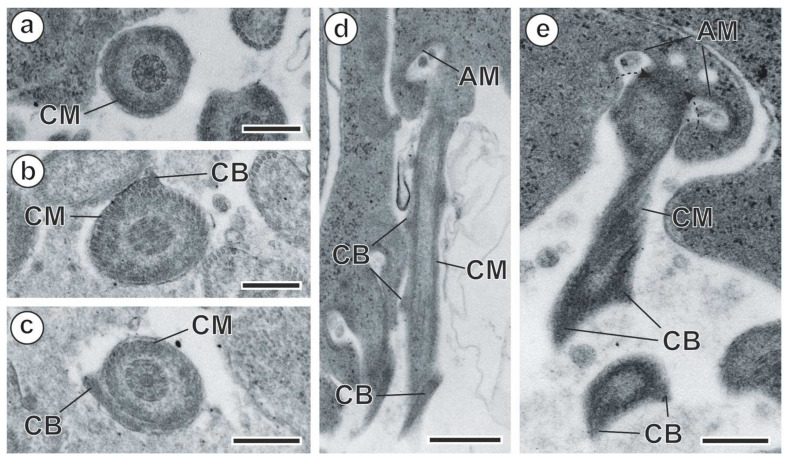
Spermiogenesis of *Meggittina gerbilli*, advanced stages. (**a**–**c**) Cross-sections show densification of the cytoplasm and formation of crest-like bodies. (**d**,**e**) Longitudinal sections of the final stage of spermiogenesis. (AM) arching membranes; (CB) crest-like bodies; (CM) cortical microtubules. Scale bars: (**a**–**c**,**e**) = 300 nm, (**d**) = 500 nm.

In *M. gerbilli*, spermiogenesis begins with the formation of a differentiation zone in the spermatid. This differentiation zone is a conical area surrounded by a submembranous layer of cortical microtubules, and it is delimited by a ring of arching membranes in its basal part (Figure 4a and Figure 5a). The nucleus and two orthogonally oriented centrioles are present in the differentiation zone (Figure 4a and Figure 5a,b). A cytoplasmic extension elongates from the differentiation zone. One of the centrioles gives rise to a free flagellum that grows more or less orthogonally to the cytoplasmic extension (Figure 4b and Figure 5c,d), whereas the other centriole aborts later. At this stage of spermiogenesis, the elongation of the nucleus in the spermatid is already observed (Figure 4b and Figure 5d). Thereafter, the free flagellum rotates and becomes parallel to the cytoplasmic extension (Figure 4c and Figure 5e,f), while fusion occurs by the so-called proximodistal fusion (Figure 4d and Figure 5f,h). Both in the cytoplasmic extension before the proximodistal fusion and in the spermatid after proximodistal fusion, cortical microtubules are parallel to the hypothetical long axis of the spermatid (Figure 5f). However, progressively, the submembranous layer of the cortical microtubules becomes twisted (Figure 5g). During the final stages of spermiogenesis, a densification in the cytoplasm is observed (Figure 6a–c), and crest-like bodies are formed (Figure 4e and Figure 6b–e). Spermiogenesis finishes with the constriction of the ring of arching membranes (Figure 4e and Figure 6e) in order to liberate the spermatozoon.

### 3.2. Spermatozoon

In the spermatozoon of *M. gerbilli*, five regions (I to V) can be considered from the anterior to the posterior extremity. These five regions are distinguished by their ultrastructural characteristics.

**Figure 7:**
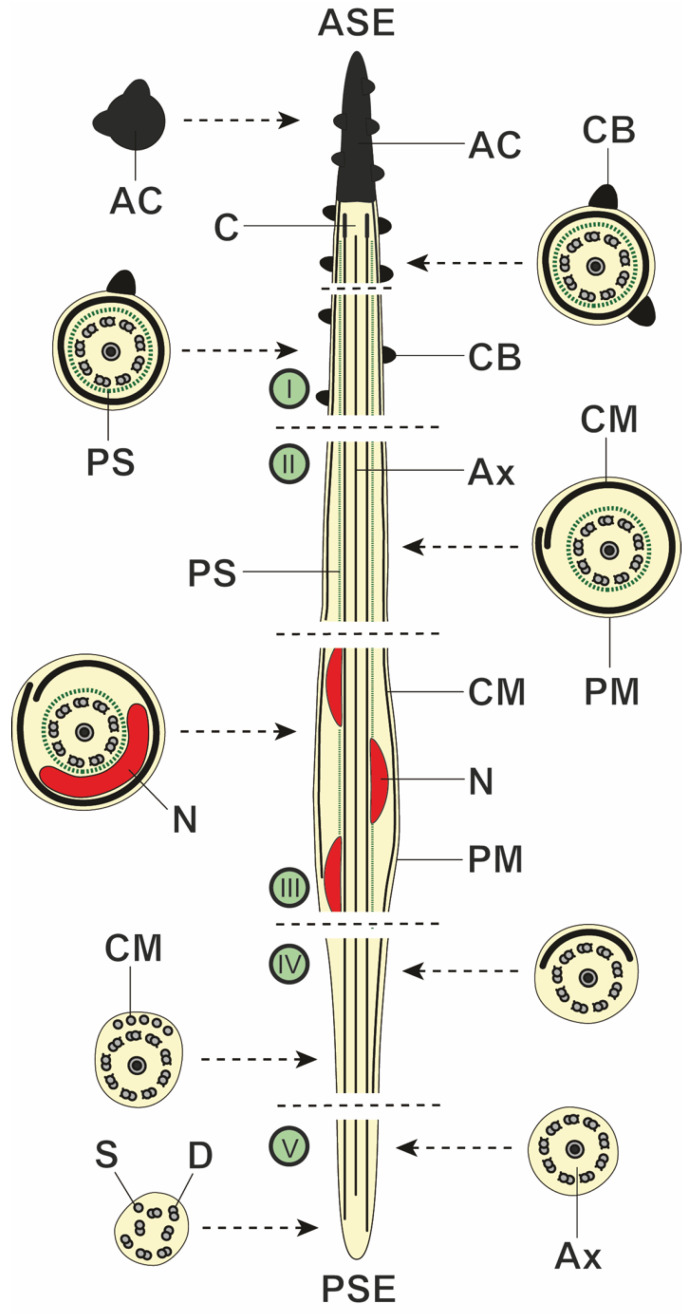
Schematic drawing showing the ultrastructural organization of the spermatozoon of *Meggittina gerbilli* into five regions (I to V). (AC) apical cone; (ASE) anterior spermatozoon extremity; (Ax) axoneme; (C) centriole; (CB) crest-like bodies; (CM) cortical microtubules; (D) doublets of axonemal microtubules; (N) nucleus; (PM) plasma membrane; (PS) periaxonemal sheath; (PSE) posterior spermatozoon extremity; (S) singlets of axonemal microtubules.

**Figure 8:**
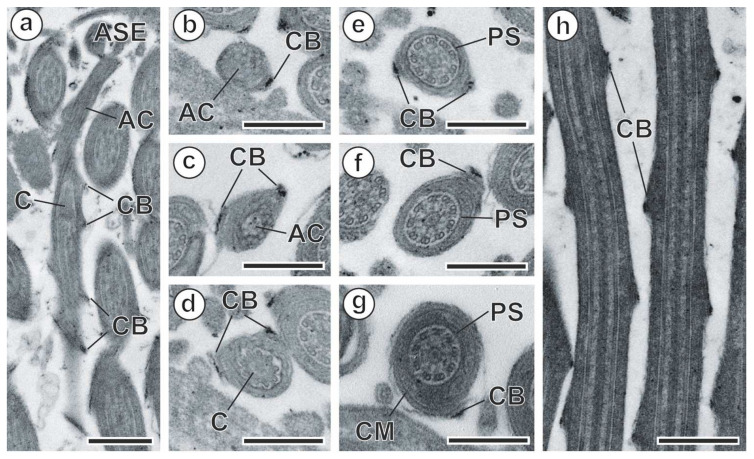
Spermatozoon of *Meggittina gerbilli*, region I. (**a**) Longitudinal section of the anterior extremity. (**b**,**c**) Cross-sections of the apical cone. (**d**) Cross-section showing the centriole. (**e**–**g**) Cross-sections showing the axoneme and crest-like bodies of the anterior, middle, and posterior parts of region I. (**h**) Longitudinal section of the middle part of region I. (AC) apical cone; (ASE) anterior spermatozoon extremity; (C) centriole; (CB) crest-like bodies; (CM) cortical microtubules; (PS) periaxonemal sheath. Scale bars: (**a**,**h**) = 500 nm, (**b**–**g**) = 300 nm.

Region I (Figure 7 and Figure 8a–h) is the anterior extremity of the spermatozoon. It has a maximum width of around 360 nm. Region I is capped with an electron-dense apical cone, which is more than 1150 nm long (Figure 7 and Figure 8a). This anterior region is also characterized by the presence of two helical crest-like bodies of unequal length (Figure 7 and Figure 8a–h). The two crest-like bodies appear at the level of the apical cone, with one appearing before the other (Figure 7 and Figure 8a–c). Thus, in the anterior part of region I, there are two crest-like bodies (Figure 8c–e), whereas in the posterior part, there is only one (Figure 8f–h). The maximum thickness of the crest-like bodies is around 70 nm, which was observed in the part of region I where only one crest-like body is present (Figure 8f,h). Other ultrastructural characteristics of this region include the presence of a periaxonemal sheath (Figure 8e–g) and a continuous and submembranous layer of cortical microtubules, which are twisted at a 30° angle in relation to the hypothetical long axis of the spermatozoon (Figure 8a,d–h).

**Figure 9:**
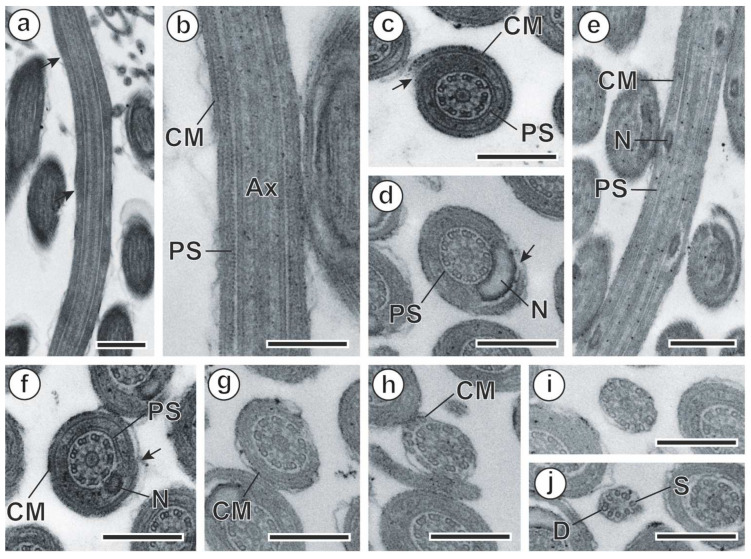
Spermatozoon of *Meggittina gerbilli*, regions II, III, IV, and V. (**a**–**c**) Longitudinal and cross-sections of region II. (**d**–**f)** Longitudinal and cross-sections of region III. (**g**,**h**) Cross-sections of region IV show the progressive disappearance of cortical microtubules. (**i**,**j**) Cross-sections of region V. (arrows) discontinuity of the layer of cortical microtubules; (Ax) axoneme; (CM) cortical microtubules; (D) doublets of axonemal microtubules; (N) nucleus; (PS) periaxonemal sheath; (S) singlets of axonemal microtubules. Scale bars: (**a**,**e**) = 500 nm, (**b**–**d**,**f**–**j**) = 300 nm.

Region II (Figure 7 and Figure 9a–c) is characterized by the disappearance of the crest-like bodies. Its maximum width is around 410 nm. Region II shows the axoneme surrounded by the periaxonemal sheath (Figure 7 and Figure 9b,c) and the spiraled cortical microtubules arranged as a discontinuous submembranous layer (Figure 7 and Figure 9a,c).

Region III (Figure 7 and Figure 9d–f) is the nuclear region of the spermatozoon. Its maximum width is around 495 nm. The nucleus is placed helicoidally around the axoneme (Figure 7 and Figure 9d,e). In the cross-sections, the nucleus appears with a round to a horse-shoe shape (Figure 9d,f). The periaxonemal sheath that surrounds the axoneme seems to be interrupted when the nucleus is present (Figure 9d–f). Finally, as in region II, in the nuclear region, the twisted cortical microtubules are arranged in a discontinuous submembranous layer (Figure 9d,f).

Region IV (Figure 7 and Figure 9g,h) has a maximum width of 400 nm and is characterized by the absence of both the nucleus and the periaxonemal sheath (Figure 7 and Figure 9g,h). Moreover, cortical microtubules progressively disappear and become parallel to the hypothetical sperm axis (Figure 7 and Figure 9g,h).

Region V (Figure 7 and Figure 9i,j) constitutes the posterior extremity of the spermatozoon. Its maximum width is around 240 nm. It is characterized by the sole presence of the axoneme (Figure 7 and Figure 9i), which progressively disorganizes into doublets and singlets (Figure 7 and Figure 9j).

## 4. Discussion

### 4.1. Spermiogenesis

In *M. gerbilli*, flagellar rotation and proximodistal fusion are the major events that characterize spermiogenesis. The process of spermiogenesis in *M. gerbilli* is quite similar to that observed in another studied catenotaeniid, *Catenotaenia pusilla* [33]. Thus, both species show a growing free flagellum that fuses proximodistally with the cytoplasmic extension. Unfortunately, no data are available concerning spermiogenesis in *Skrjabinotaenia lobata* [34], the only catenotaeniid with information on the ultrastructure of the sperm cell that belongs to the same subfamily (Skrjabinotaeniinae) as *M. gerbilli* (Table 1). In *C. pusilla*, the flagellum develops at an angle of about 45° to the cytoplasmic extension [33]. However, an angle of about 90° was observed in the present study. Świderski [16] and, posteriorly, Bâ and Marchand [20] characterized spermiogenesis in cestodes, establishing three and four models, respectively. In cyclophyllidean cestodes, two types of spermiogenesis were described: III and IV. These two models differ in the presence or absence of a proximodistal fusion [20]. The two catenotaeniids studied to date follow a “modified” type III of Bâ and Marchand. Type III was defined based on a free flagellum growing parallel to the cytoplasmic extension and, consequently, lacking flagellar rotation [20]. However, the Catenotaeniidae representatives, *C. pusilla* [33] and *M. gerbilli*, both present flagellar rotation (Table 1). Likewise, other cyclophyllideans did not fit the traditional type III spermiogenesis proposed by Bâ and Marchand [20]. These are the paruterinids *Anonchotaenia globata*, *Notopentorchis* sp., and *Triaenorhina rectangula* [28,36,37], and the taeniids *Taenia parva* and *Taenia taeniaeformis* [38,39]. Finally, a doubtful slight flagellar rotation, unsupported by the published TEM micrographs, has been mentioned in the metadilepidid *Skrjabinoporus merops* [40].

Other characteristics of spermiogenesis in *M. gerbilli* include the absence of striated rootlets associated with centrioles and the absence of an intercentriolar body. These structures are typically associated with cestode spermiogenesis types I and II, which are observed in species of other cestode orders [16,20]. However, striated rootlets or similar structures are observed in some cyclophyllideans presenting spermiogenesis types III or IV. This is the case for the dipylidiids *Dipylidium caninum*, *Joyeuxiella echinorhynchoides*, and *Joyeuxiella pasqualei*, the paruterinids *A. globata*, *Notopentorchis* sp., and *T. rectangula*, and the taeniid *T. taeniaeformis*, which present striated rootlets or vestigial striated rootlets associated with the centrioles in a type III spermiogenesis [28,36,37,39,41,42]. Moreover, these structures are also present in the anoplocephalids *Anoplocephaloides dentata*, *Gallegoides arfaai*, and *Mosgovoyia ctenoides*, which follow a type IV spermiogenesis [43,44,45].

The cytoplasm densification observed in advanced stages of *M. gerbilli* spermiogenesis could be the origin of the periaxonemal sheath or the crest-like bodies. Unfortunately, no TEM micrographs illustrating this process were obtained. Ndiaye et al. [42] described the formation of the periaxonemal sheath in *J. echinorhynchoides* (Dipydidiidae), which was initiated by cytoplasm densification in the periphery of the spermatid and showed a progressive displacement towards surrounding the axoneme.

### 4.2. Spermatozoon

Considering the spermatozoon, the Eucestoda includes species with one or two axonemes in their male gametes. Thus, sperm cells with two axonemes are present in the orders Diphyllobothriidea, Spathebothriidea, Haplobothriidea, Onchoproteocephalidea, and Trypanorhyncha [21,22,23,46,47,48,49,50,51,52,53,54,55,56,57,58,59,60]. Contrarily, spermatozoa with one axoneme are present in the orders Caryophyllidea, Cyclophyllidea, Lecanicephalidea, Nippotaeniidea, Phyllobothriidea, and Tetrabothiidea [21,22,23,26,27,28,29,30,31,32,61,62,63,64,65,66,67,68,69,70,71,72]. In the order Diphyllidea, there are some discrepancies between the spermiogenesis process and the resulting spermatozoa. During spermiogenesis, two flagella are formed from the zone of differentiation. However, paradoxically, the spermatozoon exhibits only one axoneme [21,73] due to either (i) one of the axonemes disappearing at the end of spermiogenesis or because of (ii) a marked longitudinal shift between the axonemes in the spermatozoon, making it highly difficult to observe cross-sections of an area where the two possible axonemes are simultaneously present [73]. In the particular case of *Echinobothrium euterpes*, Marigo et al. [74] described the formation of a short flagellum about 1 µm long, which was also observed after flagellar rotation and proximodistal fusion in the final stages of spermiogenesis after nuclear migration. This reduced axoneme, if present in the sperm cell, would cover a very reduced region, considering the length of the spermatozoon. In other Diphyllideans, such as *Echinobothrium affine* and *Echinobothrium harfordi*, the estimated total length of the male gamete was 200 µm [73]. In the order Onchoproteocephalidea, one axoneme was described in the spermatozoon of *Sandonella sandoni* [75], whereas two axonemes were described in the sperm cells of all of the remaining studied species [21,22,23,52,54].

In *M. gerbilli*, as in all the Eucestoda, the spermatozoon lacks mitochondrion, which was postulated as a synapomorphy for this subclass [17,21,22]. Contrarily, the representatives of the subclass Cestodaria, which includes the orders Amphilinidea and Gyrocotylidea, are the only cestodes with mitochondria in their male gametes [76,77].

The spermatozoon of *M. gerbilli* follows the type VI of Levron et al. [23]. According to these authors, type VI sperm cells are characterized by the presence of a single axoneme, crest-like bodies, spiraled cortical microtubules, periaxonemal sheath, and a spiraled nucleus. This sperm model is typical of species belonging to the families Catenotaeniidae, Dilepididae, Dipylidiidae, and Gryporhynchidae, and it is also present in species of the Anoplocephalidae [29,33,34,41,74,78,79,80,81,82,83]. Thus, the type VI spermatozoon was found in the anoplocephalids, *Mathevotaenia herpestis* and *Stilesia globipunctata* [78,79], in the catenotaeniids, *C. pusilla* and *S. lobata* [33,34], in the dilepidids, *Angularella beema*, *Anomotaenia quelea*, and *Molluscotaenia crassiscolex* [29,72,82], in the dipylidiids, *D. caninum*, *J. pasqualei*, and *J. echinorhynchoides* [41,80,81], and in the gryporhynchid, *Valipora mutabilis* [83]. Considering the available data, only catenotaeniids, dipylidiids, and the anoplocephalid *M. herpestis* present the same pattern of spermiogenesis (III) and the same spermatozoon (VI) model.

Crest-like bodies are helical cordons that are present in the anterior extremity of the spermatozoon of most cestodes. The number of crest-like bodies varies between 1 and 12 [21,32,72,84], although only 1 or 2 crest-like bodies have commonly been found in the species studied so far, including *M. gerbilli*, with two crest-like bodies. However, some species exhibit a higher number, particularly those belonging to the Cyclophyllidean family Hymenolepididae. These species are the Onchoproteocephalidea *Nomimoscolex* sp. (with three crest-like bodies) [85], the Anoplocephalidae *Aporina delafondi* (with five) and *Sudarikovina taterae* (with seven) [86,87], and the Hymenolepididae *Cladogynia serrata* and *Rodentolepis microstoma* (with six), *Echinocotyle dolosa* and *Rodentolepis straminea* (with eight), *Hymenolepis sulcata* and *Rodentolepis myoxi* (with nine), *Rodentolepis fraterna* (with 10), and *Rodentolepis nana* (with 12) [88]. On the other hand, crest-like bodies are absent in cestodes of the orders Amphilinidea, Gyrocotylidea, Caryophyllidea, Diphyllobothriidea, Haplobothriidea, Spathebothriidea, and Trypanorhyncha [21,22,23].

The spiraled pattern of the cortical microtubules was postulated as a synapomorphy for the Cyclophyllidea and Tetrabothriidea [17,21,22]. However, species of the cyclophyllidean family Mesocestoididae possess parallel cortical microtubules in the spermatozoon [89,90]. In the remaining cyclophyllideans, the cortical microtubules in the spermatozoon are twisted at an angle between 10 and 60° [32,72,84]. In the Catenotaeniidae, this angle varies between 30 and 40° (see Table 1), similar to numerous species of other cyclophyllidean families [32,72,84].

## 5. Conclusions 

Spermiogenesis of *Meggittina gerbilli* follows Bâ and Marchand’s type III, which is characterized by a proximodistal fusion of a single flagellum with a cytoplasmic extension. However, the observed flagellar rotation in *M. gerbilli* constitutes a characteristic that was also mentioned in *Catenotaenia pusilla*, the other catenotaeniid in which spermiogenesis has been studied to date. Spermiogenesis type III in these two catenotaeniids can be distinguished from spermiogenesis type II by the absence of striated rootlets and intercentriolar body. The spermatozoon of *M. gerbilli* is of Levron et al.’s type VI, characterized by having one axoneme, a periaxonemal sheath, crest-like bodies, twisted cortical microtubules, and a spiraled nucleus. Moreover, the mature spermatozoon in the three studied catenotaeniids have two crest-like bodies of unequal length and similar maximum thicknesses (between 60 and 80 nm). Thus, despite the scarce ultrastructural data on this family, the currently available ultrastructural results emphasize the similarities in the spermatological characteristics of the Catenotaeniidae.

## Figures and Tables

**Table 1:** Main ultrastructural characteristics of spermiogenesis and the spermatozoon in Catenotaeniidae species.

Subfamily,	Spermiogenesis					Spermatozoon Characteristics									
Species, and	Characteristics						CB								
Reference	Type ^1^	PF	FR	SR	IB	Type ^2^	n	T	L	AC	CM	N	DG	PS	IW
Catenotaeniinae															
*Catenotaenia pusilla* [33]	III	+	+ (~45°)	–	–	VI	2	75	≠	1750 × 225	40°	Sp	–	+	–
Skrjabinotaeniinae															
*Meggittina gerbilli* Present study	III	+	+ (~90°)	–	–	VI	2	70	≠	>1150	30°	Sp	–	+	–
*Skrjabinotaenia lobata* [34]	?	?	?	?	?	VI	2	60–80	≠	2500 × 200	40°	Sp	–	+	–

All measurements are given in nm. ^1^ according to Bâ and Marchand [20]; ^2^ according to Levron et al. [23]; (AC) apical cone, length × width; (CB) crest-like bodies; (CM) angle of cortical microtubules; (DG) dense granules; (FR) flagellar rotation; (IB) intercentriolar body; (IW) intracytoplasmic walls; (L) length of crest-like bodies; (n) number of crest-like bodies; (N) nucleus; (PF) proximodistal fusion; (PS) periaxonemal sheath; (Sp) spiraled; (SR) striated rootlets; (T) maximum thickness of crest-like bodies; (+) presence; (–) absence; (≠) unequal; (?) unknown data.

## Data Availability

The data presented in this study are available upon request from the corresponding author. The data is not publicly available due to internal laboratory policy.
